# Structural and electronic switching of a single crystal 2D metal-organic framework prepared by chemical vapor deposition

**DOI:** 10.1038/s41467-020-19220-y

**Published:** 2020-11-02

**Authors:** F. James Claire, Marina A. Solomos, Jungkil Kim, Gaoqiang Wang, Maxime A. Siegler, Michael F. Crommie, Thomas J. Kempa

**Affiliations:** 1 Department of Chemistry, Johns Hopkins University, Baltimore, MD USA; 2 Department of Physics, University of California Berkeley, Berkeley, CA USA; 3 Materials Sciences Division, Lawrence Berkeley National Laboratory, Berkeley, CA USA; 4 Kavli Energy NanoSciences Institute at the University of California Berkeley and the Lawrence Berkeley National Laboratory, Berkeley, CA USA; 5 Department of Materials Science and Engineering, Johns Hopkins University, Baltimore, MD USA

**Keywords:** Metal-organic frameworks, Design, synthesis and processing

## Abstract

The incorporation of metal-organic frameworks into advanced devices remains a desirable goal, but progress is hindered by difficulties in preparing large crystalline metal-organic framework films with suitable electronic performance. We demonstrate the direct growth of large-area, high quality, and phase pure single metal-organic framework crystals through chemical vapor deposition of a dimolybdenum paddlewheel precursor, Mo_2_(INA)_4_. These exceptionally uniform, high quality crystals cover areas up to 8600 µm^2^ and can be grown down to thicknesses of 30 nm. Moreover, scanning tunneling microscopy indicates that the Mo_2_(INA)_4_ clusters assemble into a two-dimensional, single-layer framework. Devices are readily fabricated from single vapor-phase grown crystals and exhibit reversible 8-fold changes in conductivity upon illumination at modest powers. Moreover, we identify vapor-induced single crystal transitions that are reversible and responsible for 30-fold changes in conductivity of the metal-organic framework as monitored by in situ device measurements. Gas-phase methods, including chemical vapor deposition, show broader promise for the preparation of high-quality molecular frameworks, and may enable their integration into devices, including detectors and actuators.

## Abstract (web summary)

The realization of advanced devices from metal-organic frameworks requires the preparation of uniform and crystalline thin-films of these materials. Here the authors use chemical vapor deposition to grow single-crystal frameworks that exhibit a reversible structural transition and large conductivity response.

## Introduction

Metal–organic frameworks (MOFs) are versatile materials with tunable architectures and significant pore volumes. Many applications take advantage of the ability of MOFs to store gases and ions efficiently and separate chemical products selectively^1,2^. Recently, there has been a burgeoning interest in developing new device concepts based on metal–organic frameworks with active or responsive properties in order to enable switches, sensors, and actuators^3–8^. Two criteria are necessary to realize such devices. First, it is necessary to design MOFs whose structures and properties can be manipulated by external stimuli^9,10^. Many studies have focused on eliciting mechanical motion or a magnetic response from molecular crystals and polymers subjected to optical or thermal stimuli. Though intriguing, many of these responses manifest only at cryogenic temperatures or exhibit slow switching behavior, thereby complicating use of these materials in practical devices^9^. Second, one must develop synthetic methods capable of preparing and seamlessly integrating high-quality crystalline framework materials into devices^11–14^. The development of conductive MOFs through judicious selection of metal nodes, incorporation of redox-active ligands, and inclusion of guest species has resulted in conductivity values as high as 2500 S cm^–1^ ^15–22^. Nevertheless, the integration of many candidate MOFs into high performance active devices has been challenging partly due to their poor crystallization characteristics and low stabilities over time. Moreover, concerns over chemical contamination, contact corrosion, and other incompatibilities underscore the issues associated with relying on solution-based methods for integration of MOFs with microelectronic devices.

Gas-phase deposition methods are a viable alternative for MOF crystal growth with the potential to overcome some of the aforementioned challenges related to solution-based approaches^23–25^. Used extensively for synthesis of thin films and low-dimensional materials such as graphene, transition metal dichalcogenides, and semiconductor nanowires, gas-phase synthesis was adapted for growth of MOF materials as recently as 2013^26^. Since then, researchers have demonstrated the multi-step molecular layer deposition (MLD) of films of UiO-66^27^ and copper (II) terephthalate^28^, and the conversion of zinc-oxide films to ZIF-8 upon exposure to 2-methylimidazole vapors^29^. While these studies mark notable progress in the use of gas-phase methods for MOF deposition, many challenges remain. First, the multi-step reactions prevalent in these studies increase synthetic overhead and require careful management of intermediates, byproducts, and multiple processing conditions. Second, the coordinated frameworks obtained from these reactions are either amorphous or polycrystalline, and, in many cases, require conversion from a bulk phase. Finally, though often motivated by the desire to incorporate these materials in devices, these studies rarely report device transport measurements.

**Fig. 1:**
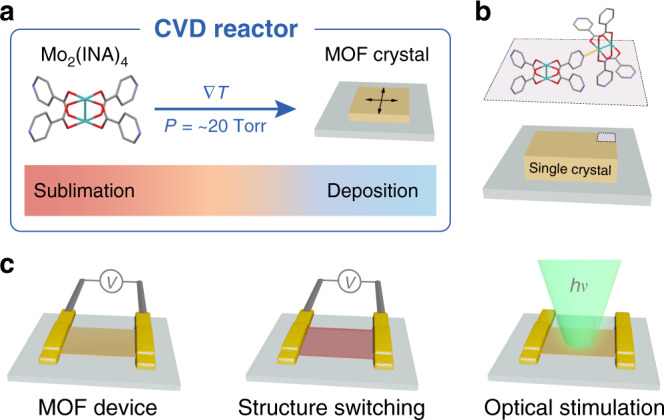
Gas-phase synthesis of large-area MOF crystals and their integration into devices. **a** Schematic outlining the process for CVD growth of high-quality MOF single crystals with minimal defects and single-crystal character. A MOF precursor, such as Mo_2_(INA)_4_, sublimes under vacuum, is swept by a carrier gas along a thermal gradient, and deposits at lower temperatures to form high-quality MOF single crystals on a range of substrates. **b** Concerted deposition and coordination of Mo_2_(INA)_4_ clusters leads to single MOF crystals covering large areas of the substrate. **c** The large-area single crystals are integrated into devices (left), which are used to monitor the electronic response of the MOF to induced structural changes (middle) and to optical stimulation (right).

In this study we use chemical vapor deposition (CVD) to prepare MOF single crystals and then demonstrate reversible switching of their structure and conductivity. The MOF crystals are comprised of Mo_2_(INA)_4_ (INA: isonicotinate) paddlewheel clusters, which serve as secondary building units and have been shown to assemble from the solution-phase into a number of topologies, including molecular chains and two-dimensional (2D) lattices^30,31^. These paddlewheel clusters resemble a broader class of di-nuclear complexes containing multiply-bonded transition metals^32–36^. Moreover, these clusters are excellent candidate building blocks of an optoelectronically responsive MOF owing to the fact that they exhibit Class III Robin-Day mixed valency and a diversity of optical transitions^37,38^. Notably, we demonstrate the sublimation and concomitant deposition of Mo_2_(INA)_4_ as thin single crystals on glass substrates under high-vacuum CVD conditions (Fig. 1a). This single step reaction involves the deposition and coordination of neighboring clusters to yield crystalline extended solids (Fig. 1b). The exceptional uniformity and quality of the as-grown MOF crystals facilitates their integration and fabrication into active and responsive single-crystal devices (Fig. 1c). To demonstrate the underlying responsiveness of the Mo_2_(INA)_4_ MOF, we monitor in situ the conductivity change of the single crystal device as the MOF undergoes structural switching and optical excitation (Fig. 1c). Collectively, these data represent the first demonstration of structural and electronic switching behavior within a single-crystal device comprised of a MOF prepared solely through gas-phase methods.

## Results and discussion

### Chemical vapor deposition of crystalline metal–organic framework

**Fig. 2:**
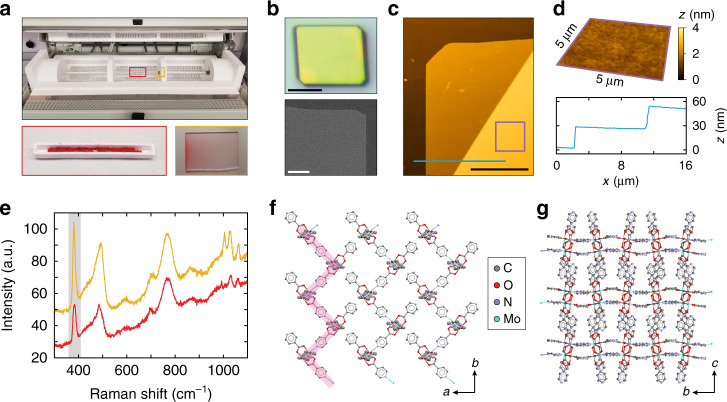
High-quality Mo_2_(INA)_4_ MOF single crystals from gas-phase synthesis. **a** Top: Photograph of the quartz tube reaction chamber of our CVD system. The reaction chamber is divided into three independent thermal zones. Reactants flow from left to right through the quartz tube. The red and yellow boxed regions denote the positions of the precursor and deposition substrate, respectively, during the reaction. Bottom left: Photograph of the ceramic crucible containing Mo_2_(INA)_4_ powder. Bottom right: Photograph of a red film of **1** deposited on a glass substrate. The left-hand side of the substrate is nearest the precursor boat. **b** Top: Optical image of a single deposit of **1**. Scale: 10 μm. Bottom: Scanning electron micrograph of a corner of one deposit of **1**. Scale: 1 µm. **c** 2D AFM topographical map of a single thin deposit of **1**. The purple box and blue line denote approximate regions of interest investigated further in (**d**). A 10 µm × 10 µm region of this AFM map was used to calculate the root mean square surface roughness reported in the text. Scale: 10 μm. **d** Top: 3D AFM topographical map encompassing the 25 µm^2^ area annotated by the purple box in (**c**). These data exemplify the exceptionally low surface roughness of the CVD-grown deposits. Bottom: AFM height profile along the path annotated by the blue line in (**c**). **e** Raman spectra of the Mo_2_(INA)_4_ powder precursor (red) and of a single deposit of **1** (yellow). The gray band highlights the prominent peak at 383 cm^–1^ attributable to $\tilde \nu$(Mo–Mo)^33^. **f** Crystal packing of **1** viewed down the *c* axis. The pink line highlights one of the 1D zig-zag coordinated chains. **g** Crystal packing of **1** viewed down the *a* axis showing its layered structure. Hydrogen atoms have been omitted for clarity. Displacement ellipsoids are given at 50% probability.

We first assessed the potential to grow singly crystalline MOFs using CVD. At a reactor pressure of 20 Torr, Mo_2_(INA)_4_ undergoes sublimation (at 325 °C) and concomitant deposition from the gas phase to form a light red coating on glass substrates (Fig. 2a). Optical images and scanning electron micrographs reveal that this coating consists of many individual rectangular deposits having faceted edges and a mean projected area of 96 µm^2^ (Fig. 2b, Supplementary Fig. 1). Extensive growth studies have identified that temperature and pressure conditions of 300–375 °C and 20–40 Torr, respectively, routinely yield near complete substrate coverage with uniform deposits having 10–50 µm edge lengths and thicknesses in the hundreds of nanometers (Supplementary Fig. 2). Performing the reaction at slightly higher temperatures or at lower pressures yields polycrystalline films. In an effort to synthesize thinner deposits, we established in our CVD reactor a gradual negative thermal gradient extending 11 cm from the site of sublimation to the site of deposition by shifting the substrate 2 cm further downstream from the precursor. Atomic force microscopy (AFM) of the products of a reaction conducted under these conditions shows that the individual deposits are ~20 nm thick and exceptionally smooth, with a measured root mean square surface roughness of *R*_*q*_ = 2.9 nm over a 100 μm^2^ area (Fig. 2c, d). Additional AFM data identify discrete steps at the edges of the deposits, suggesting a layered structure (Supplementary Fig. 3). Raman spectra acquired on a solid powder of Mo_2_(INA)_4_ before CVD synthesis and on a substrate coated with the aforementioned deposits after CVD growth are largely identical. Notably, a prominent peak at 383 cm^–1^, which is attributable to the $\tilde \nu$(Mo–Mo) mode^33^ associated with the quadruple-bonded di-Mo core of the Mo_2_(INA)_4_ cluster, is evident in both spectra (Fig. 2e). These Raman data suggest that the deposits after CVD synthesis are comprised of intact Mo_2_(INA)_4_ clusters.

The X-ray crystal structure of a single CVD-grown deposit reveals a uniform crystal comprised of “zig-zagging” one-dimensional (1D) chains of partially-coordinated Mo_2_(INA)_4_ paddlewheels (Fig. 2f, Supplementary Fig. 4). Henceforth, crystals exhibiting this CVD-grown structure will be referred to as **1**. This crystal structure is consistent with our reported Raman data, which showed that the deposits are comprised of intact Mo_2_(INA)_4_ paddlewheel clusters. Mo–Mo bond lengths are measured at 2.1143(4) Å throughout the structure. These bond distances are consistent with those previously reported in quadruple-bonded Mo_2_ cores^31,39^. Individual 1D chains propagate along the *b*-axis, while parallel chains assemble along the *a* axis. Parallel 1D chains form sheets, which stack in the *c* direction, are spaced 2.835 Å apart, and are offset by half a unit cell (Fig. 2g). Superimposed sheets are separated by a larger distance of 9.617 Å. The as-grown crystals observed by optical microscopy and AFM are comprised of these layered crystalline sheets, and the crystals appear to have their major (002) face oriented parallel to the substrate (Supplementary Fig. 5). Notably, though the Mo and N atoms of adjacent 1D chains do not engage in coordination bonds, the distance between these atoms (Fig. 2f, Supplementary Fig. 6) is such that inter-chain binding may be feasible under appropriate conditions to yield a new framework.

### Reversible structural switching of framework

**Fig. 3:**
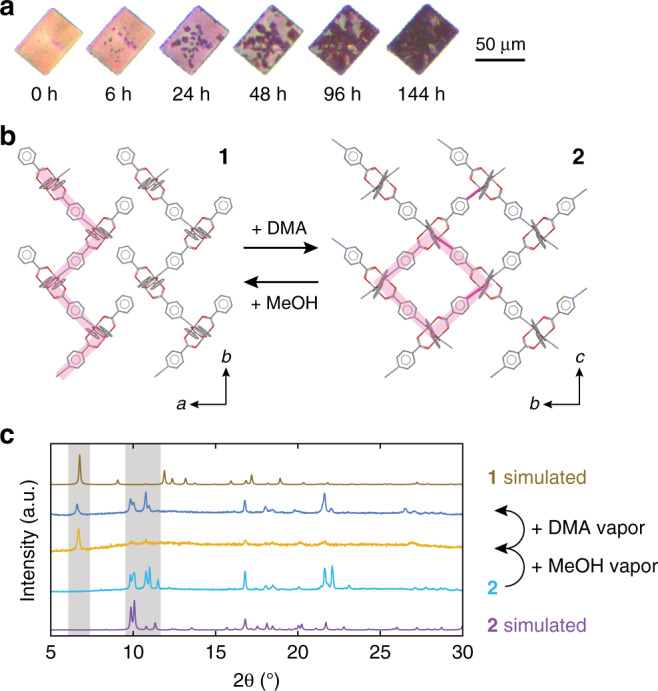
Reversible structural switching between 1 and 2. **a** Photographs of a single crystal of **1** exposed over time to dimethylacetamide (DMA) vapor. **b** Single-crystal structures of **1** (left) and of **2** (right) showing how exposure to DMA and methanol can facilitate interconversion between the two structures. Solvent molecules have been omitted for clarity. **c** Simulated and experimental powder XRD scans of bulk samples of **1** and **2** used to demonstrate reversible conversion between the MOF structures.

We next explored the possibility of inducing single-crystal structural transitions in our molecular framework. A large single crystal of **1** was monitored in the presence of dimethylacetamide (DMA) vapor over the course of 7 days. After 6 h in the presence of DMA vapor, optical images reveal the appearance of red regions within the initially yellow-orange crystal (Fig. 3a). The size of these red regions grows under continued exposure to DMA vapor, and nearly complete conversion to a dark red crystal is observed in 6 days. X-ray structural analysis of **1** exposed to DMA vapor reveals a crystal comprised of layered 2D lattices of fully-coordinated Mo_2_(INA)_4_ clusters (Fig. 3b, Supplementary Fig. 7). Henceforth, crystals exhibiting this 2D MOF structure will be referred to as **2**. **2** crystallizes in the P2/c space group, exhibits body diagonals of 14.000 and 13.004 Å, and interplanar stacking distances of 7.790 Å. Notably, **2** exhibits similar packing and unit cell dimensions (Supplementary Fig. 8) to our previously reported 2D MOF, which was prepared solely through solution-phase methods^31^. While the twisting of coordinated Mo_2_(INA)_4_ clusters along the 1D chains in **1** likely prevents Mo–N bond formation between adjacent parallel 1D chains, the introduction of DMA may facilitate inter-chain binding to form **2**, whose Mo–N bond lengths measure 2.568(8) and 2.559(6) Å and are similar to those in our previously reported 2D MOF. It is possible that entry of DMA into the crystal lattice accommodates a structural adjustment sufficient to allow Mo–N bond formation across adjacent 1D chains, ultimately leading to formation of the fully-coordinated 2D lattice with disordered solvent molecules situated within square sub-cells.

We subsequently assessed the reversibility of structural transitions involving our as-grown and converted MOFs. Bulk samples of **2** were prepared to facilitate conversion trials. The powder X-ray diffraction (pXRD) scan of a bulk sample of **2** (Fig. 3c, light blue line) contains the same principal peaks between 9.7° and 12.4° as the pXRD scan of **2** obtained through routine simulation from the single-crystal structure of **2** (Fig. 3c, purple line). These data verify that bulk quantities of **2** are representative of the single-crystal structure of **2**. Subsequently, a 10 mg sample of **2** was exposed to methanol vapor for 48 h. A dominant peak at 6.7° is apparent in the pXRD scan of this methanol exposed sample (Fig. 3c, yellow line). A peak at this same 2*θ* angle is present in the pXRD scan of **1** obtained through routine simulation from the single-crystal structure of **1** (Fig. 3c, brown line). These data suggest that **2** converts to **1** upon exposure to methanol. The 6.7° peak likely corresponds to the aforementioned (002) plane of **1**, because this is the major crystal face in the highly oriented CVD-grown crystals (Supplementary Fig. 5). Heat capacity measurements on dried samples of **1** show no additional transitions between 2 and 300 K (Supplementary Fig. 9). Finally, this same sample was exposed to DMA vapor for 48 h and its pXRD scan was acquired (Fig. 3c, dark blue). The peaks within this scan are similar (traces of **1** remain) to those found in the simulated scan of **2** after it was first converted from **1** by exposure to DMA. The small differences between 10° and 12° and emergent peaks near 22° in the two pXRD scans for the 2D phases (Fig. 3c, light blue and dark blue) can be attributed to minor structural differences arising from different and possibly incomplete DMA incorporation during separate conversion cycles (Supplementary Fig. 10). Together, these data show that **2** can be converted to **1** through exposure to methanol and then back to **2** through exposure to DMA, thereby showing the reversible and cyclic structural transitions our Mo_2_(INA)_4_ MOF system is capable of.

### Electronic response during structural switching

**Fig. 4:**
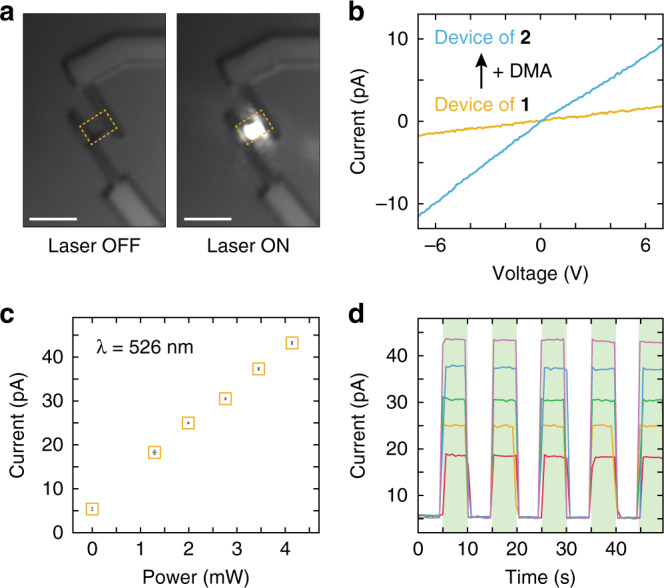
Stimuli responsive conductivity of Mo_2_(INA)_4_ single-crystal devices. **a** Optical images collected during measurement of a device fabricated on a single crystal of **1**. The crystal boundary is highlighted by the dashed gold box and the bright diffraction-limited laser spot is evident in the right image. Scale: 5 µm. **b** Current–voltage characteristics of a single-crystal device of **1** (yellow) and of a single-crystal device of **2** (blue) prepared through exposure to DMA. **c** Photocurrent generated by a single-crystal device of **1** when illuminated with a 526 nm laser at 1.29, 1.99, 2.76, 3.45, and 4.14 mW. The device source-drain bias was held constant at 20 V. Error bars denote standard deviation. **d** Photocurrent switching behavior of a single-crystal device of **1** subjected to periodic laser illumination at 5-s intervals (green highlighted regions). Device switching behavior was assessed at 1.29 mW (red), 1.99 mW (orange), 2.76 mW (green), 3.45 mW (blue), and 4.14 mW (purple).

Our identification of reversible structural transitions inspired us to look for other examples of switching behavior in our MOF. To explore the responsive properties of our MOF further, we undertook fabrication of high-quality devices on single crystals of **1**. As noted above, the as-grown crystals are singly crystalline, are absent of solvent, and have uniformly flat surfaces. Together, these features facilitate the fabrication of high-quality devices which permit the measurement of the intrinsic transport properties of the molecular framework. Large single crystals of **1** were transferred via mechanical exfoliation to a Si_3_N_4_/SiO_2_/Si substrate. To make a device, two Au/Ti contacts were selectively fabricated atop either side of a transferred crystal through the use of electron-beam lithography and thermal evaporation (Fig. 4a). An AFM line profile of the resulting device confirms that the crystal is 247-nm thick and has a uniform topography (Supplementary Fig. 11). Current–voltage (*I*–*V*) measurements performed on the single-crystal device of **1** reveal a linear dependence of drain current on source-drain voltage with an extracted conductivity value of 2.25 × 10^–8^ S cm^–1^ (Fig. 4b, Supplementary Fig. 11). This value is comparable to conductivity values reported for electrically conductive MOFs and represents one of the few examples of transport measurements taken at the level of a single MOF crystal^11,15,40–43^. Notably, when single-crystal devices of **1** were exposed to the conditions previously used to convert **1** to **2**, a higher conductivity value of 6.22 × 10^–7^ S cm^–1^ was observed (Fig. 4b, Supplementary Fig. 11). This nearly 30-fold increase in conductivity is likely due to the significant increase in carrier percolation paths in going from a partially- to a fully coordinated network of Mo_2_(INA)_4_ clusters. The occurrence of a large conductivity switch during absorption of DMA vapor suggests the promise of our MOF platform in chemical sensing. To the best of our knowledge, these data represent the first in situ measurements of electronic property change in a single-crystal MOF undergoing a structural transition in response to an external stimulus.

### Electronic response during optical stimulation

To further elucidate the responsive characteristics of our MOF devices, we assessed their response to optical illumination. We irradiated a single-crystal device of **1** with the output of a 526 nm diode laser and measured its current response (at *V*_SD_ = 20 V) as a function of laser power (Fig. 4c). When not illuminated and kept in a rigorously darkened enclosure, the MOF crystal device produced a “dark” current of 5.39 pA. When illuminated, the device produced a current with linear dependence on laser power, with irradiation at 4.14 mW yielding an 8-fold larger current than the “dark” current. Moreover, periodic illumination of the device at 5-s intervals yielded reversible cycling of photocurrent at laser powers of 1.29, 1.99, 2.76, 3.45, and 4.14 mW (Fig. 4d). The device photocurrent output for a particular laser power did not deviate by more than 2.7% between illumination cycles and the device returned to within 6.4% of its “dark” current value whenever not illuminated. In a study of a distinct but related framework comprised of Mo_2_(INA)_4_ clusters, we found evidence of dimer-like metal-to-ligand charge transfer (MLCT) states at energies of 2.19 eV (566 nm)^30^. The photocurrent produced by our material may arise from excitation of such MLCT states, which are created through a lifting of the degeneracy of the single cluster orbitals. These data establish the robust photocurrent switching properties of our single-crystal MOF device and suggest the promise of this and related MOF systems in photodetection applications.

### Scanning tunneling microscopy analysis of framework assembly

**Fig. 5:**
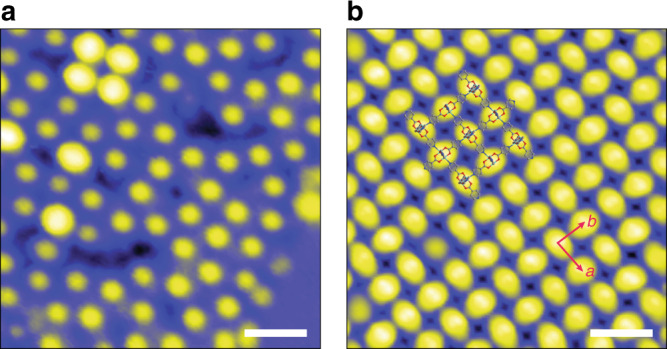
STM topography images of a Mo_2_(INA)_4_ monolayer on a Au(111) surface. **a** STM image (bias *V* = 0.05 V, tunneling current *I* = 5 pA) of Mo_2_(INA)_4_ clusters deposited on Au(111) before annealing. Scale: 2.0 nm. **b** STM image (*V* = 2 V, *I* = 10 pA) of a 2D Mo_2_(INA)_4_ MOF monolayer on Au(111) after annealing (crystal structure **2** is superimposed). Scale: 2.0 nm.

Finally, we sought to inspect the molecular-level assembly of Mo_2_(INA)_4_ from the gas phase by scanning tunneling microscopy (STM). A single-layer 2D Mo_2_(INA)_4_ MOF was grown on a Au(111) surface in ultra-high vacuum by thermal deposition and annealing. An STM topographical map of a Au surface containing deposited Mo_2_(INA)_4_ clusters before annealing shows that the clusters are randomly distributed and are present in different orientations, as evidenced by the different intensities they elicit in the STM map (Fig. 5a). After annealing, the Mo_2_(INA)_4_ clusters form a highly-ordered monolayer characterized by alternating 90°-rotated oval features that form a square lattice with associated lattice constants *a* = *b* = 10.8 ± 0.4 Å (Fig. 5b, Supplementary Fig. 12). Notably, the distance between the centroids of two neighboring clusters (centroid here is defined as the midpoint of the Mo–Mo bond axis) in the crystal structure of **2** ranges between 10.535 and 10.551 Å. The order and spacing of the features in the STM map are consistent with the arrangement of Mo_2_(INA)_4_ clusters in the crystal structures of **1** and **2**, and with the distance between Mo_2_(INA)_4_ clusters in the structure of **2**, respectively. These data suggest that growth at elevated temperatures is sufficient to facilitate the organization of randomly deposited Mo_2_(INA)_4_ clusters into our reported MOF phases. Facile deposition and ordering of individual Mo_2_(INA)_4_ clusters under gas-phase synthesis conditions reinforces the potential for use of CVD methods in the preparation of flat, uniform, and single-molecule thick MOFs suitable for detailed study.

We have demonstrated the growth of high-quality single crystals of a responsive MOF using gas-phase methods. We show that the paddlewheel cluster, Mo_2_(INA)_4_, sublimes as a molecular unit to form an extended framework in the absence of solvent and is capable of assembly into a truly two-dimensional (2D), single-molecule thick MOF. Moreover, we show that the crystal structure of this material can be reversibly switched to and from a layered 2D framework upon exposure to solvent vapors. Devices fabricated from single crystals of **1** show appreciable electronic conductivity. Notably, we measured in situ a 30-fold increase in conductivity as a single-crystal device underwent conversion to a 2D topology. Furthermore, we found a significant and cyclable photocurrent response for single-crystal devices. We also note that our MOFs exhibit concurrent responses in their conductivity and structure at room temperature and can therefore be distinguished from many of the previously studied responsive molecular crystal and polymer systems. We foresee broader use of gas-phase methods, including CVD, for the preparation of high-quality molecular frameworks (e.g., MOFs and COFs), and the prospect for facile integration of these diverse materials into devices, including sensors, actuators, and detectors.

## Methods

### CVD reactor

Our homebuilt CVD system is a versatile quartz tube hot-wall reactor design with a manifold of mass flow controllers (MKS Instruments: GM50A series MFCs) and a closed-loop pressure control system (MKS Instruments: 640B pressure controller). The manifold and pressure control circuit are both operated through custom LabView scripts running on a PC. The furnace (Thermo Scientific-Lindeberg Blue M – 3 zone) has three independently controllable zones, each of which measures 25 cm in length and can reach temperatures of 1200 °C. A 400 °C temperature differential can be maintained between adjacent zones through the use of thermal inserts. A high-vacuum pump (Leybold: LV80 screw pump) is used to evacuate our CVD system to a base pressure of 0.01 mTorr and is able to safely manage any toxic or pyrophoric effluent. The metal sealed GM50A series mass flow controllers on our CVD reactor permit highly accurate (1% set-point accuracy) flow control and are accompanied by NIST traceable calibration sheets. Prior to all CVD reactions, we thoroughly washed and then performed bake out of quartz tubes (Quartz Plus: 22 mm inner diameter, 25.4 mm outer diameter) at 600 °C for 1 h under a 10 sccm N2 flow.

### CVD synthesis and methods

Crystalline powder Mo_2_(INA)_4_ (0.010 g) was added to a ceramic boat and this boat was placed in a quartz tube (see the “Methods” section) along with a glass or SiO_2_/Si deposition substrate. Substrates were cleaned by sonication in acetone and IPA and then dried with compressed air. When the quartz tube was placed in the three-zone furnace, the boat containing Mo_2_(INA)_4_ was centered in the middle zone and the deposition substrate was placed 9 cm downstream of the precursor boat and aligned with a thermal insulator between the center and downstream zones. The system was degassed to 1 × 10^–5^ Torr. The system was held at 20 Torr with a 20 sccm N_2_ flow. The middle zone was heated to 325 °C at a rate of 15°/min and then held at 325 °C for 15 min. The set point for the temperature controller operating the downstream zone, which contains the substrate, was set to room temperature. Based on independent calibration runs and measurements, we estimate the temperature inside the quartz tube to be ~40 °C higher than the controller set-point value and the region containing the substrate to be ~100 °C cooler than the middle zone containing the precursor. From these data points we estimate the substrate temperature to be ~265 °C. After the initial 15 min of reaction time, the reactor was allowed to cool slowly over 20 min to 200 °C while maintaining 20 Torr. Finally, to rapidly terminate the reaction, the furnace lid was propped open, thereby allowing the quartz tube to cool by 100 °C within ~1 min, and the reactor pressure was raised to atmosphere.

For reactions yielding thinner deposits, the only modification to these aforementioned reaction conditions was the placement of the substrate 11 cm downstream of the precursor. The optical images in Fig. 2 and Supplementary Fig. 1 are of crystals synthesized with the substrate positioned 9 cm from the precursor boat. The SEM and AFM images in Fig. 2 are of crystals synthesized with the substrate positioned 11 cm from the precursor boat.

### AFM methods

Atomic force microscopy was performed in tapping mode using a Molecular Imaging PicoScan Controller. Aluminum reflex coated Si AFM probes were used. Data were processed and analyzed using Gwyddion 2.49 software.^44^

### OM methods

Optical images were obtained using a Zeiss Axio Scope.A1 microscope interfaced with a Zeiss Axiocam 105 color microscope camera (5 megapixel resolution). CVD-grown crystals of Mo_2_(INA)_4_ were imaged directly on growth substrates and analyzed using ZEN software.

### Powder X-ray diffraction

Powder X-ray diffraction data were collected on a Bruker D8 Focus diffractometer using a Cu X-ray source (Kα = 8.04 keV, 1.5406 Å). The step size for a 5–60°, 4 min scan is 0.018585749° using a LynxEye detector. The exact scan time is 4 min and 10 s, using 2961 steps at 0.08 s/step. Measurements were performed at room temperature. The simulated pXRD patterns shown in Fig. 3 and Fig. S5 (blue trace) were calculated using the Mercury 3.10 powder calculation function. The simulated wavelength was set to 1.54056 Å and the peak shape was calculated for a FWHM (2*θ*) equal to 0.1.

### Single-crystal X-ray diffraction

Individual single-crystal samples were isolated from the substrate on which they were grown/formed via an oil-lubricated Kapton loop. All reflection intensities were measured at 175(2) K using a SuperNova diffractometer (equipped with Atlas detector) with Cu Kα radiation (*λ* = 1.54178 Å) under the program CrysAlisPro (Version CrysAlisPro 1.171.39.29c, Rigaku OD, 2017). The same program was used to refine the cell dimensions and for data reduction. The structure was solved with the program SHELXS-2014/7^45^ and was refined on *F*^2^ with SHELXL-2014/7^45^. Analytical numeric absorption correction using a multifaceted crystal model was applied using CrysAlisPro. The temperature of the data collection was controlled using the system Cryojet (manufactured by Oxford Instruments). The H atoms were placed at calculated positions using the instructions AFIX 43 with isotropic displacement parameters having values 1.2 Ueq of the attached C atoms. The structure is partly disordered. The crystallographic structure of **1** is provided in Supplementary Fig. 13.

A few attempts to collect data at 110 K were made (the crystals were flashcooled from RT to 110 K), but the data were unsatisfactory as the crystals always diffracted weakly (possibly due to some crystal damage from a solid-solid phase transition). A phase transition occurring at *ca*. 150 K was later confirmed via a set of *Cp* vs. *T* measurements. It was then decided to collect another data set at 175 K (the crystal being flashcooled from RT to 175 K). At 175 K, the crystal behaved normally, and the diffraction pattern was clean and consistent with a single crystal.

One of the four coordinated 4-pyridinecarboxylate is found to be disordered over two orientations, and the occupancy factor of the major component refines to 0.51(2). Because **a** and **b** are approximately equal in length, a check for twinning (an orthorhombic unit cell emulating a tetragonal unit cell) was done using the twin relationship 0 1 0 / 1 0 0 / 0 0 −1. The BASF scale factor refined to zero, and thus twinning could be ruled out. The crystal lattice contains some solvent accessible voids that contain some unresolved electron density. In the asymmetric unit, three peaks (Q7–Q8–Q9) ranging from 0.72 to 0.74 e^−^ Å^3^ were found in one void. As those residual electron density peaks were small in value and could not be fully interpreted (maybe some partially occupied lattice water molecule trapped in the crystal), their contribution was then removed from the final refinement using the SQUEEZE^46^ procedure.

### Single-crystal X-ray crystallography of 1 → 2

Individual single-crystal samples were isolated from the substrate on which they were grown/formed via an oil-lubricated Kapton loop. All reflection intensities were measured at 110(2) K using a SuperNova diffractometer (equipped with Atlas detector) with Cu Kα radiation (*λ* = 1.54178 Å) under the program CrysAlisPro (Version CrysAlisPro 1.171.39.29c, Rigaku OD, 2017). The same program was used to refine the cell dimensions and for data reduction. The structure was solved with the program SHELXS-2014/7^45^ and was refined on *F*^2^ with SHELXL-2014/7^45^. Analytical numeric absorption correction based on a multifaceted crystal model was applied using CrysAlisPro. The temperature of the data collection was controlled using the system Cryojet (manufactured by Oxford Instruments). The H atoms were placed at calculated positions using the instructions AFIX 33, AFIX 43, or AFIX 137 with isotropic displacement parameters having values 1.2 or 1.5 Ueq of the attached C atoms. The structure is partly disordered. The crystallographic structure of **2** is provided in Supplementary Fig. 14.

The Mo–Mo complex is found to be mostly ordered, except for one −C5H4N group that is disordered over two orientations. The occupancy factor of the major component of the disorder refines to 0.64(3).

The crystal lattice contains some amount of lattice DMA solvent molecules, and the ratio (Mo–Mo complex):DMA is 1:2. There are four crystallographically independent lattice DMA solvent molecules in the asymmetric unit, but their occupancy factors are all constrained to 0.5 as those solvent molecules are located at sites of twofold axial symmetry. One of the four lattice DMA solvent molecule is heavily disordered over 3 orientations, and no anisotropic refinement was performed for those atoms (i.e., C5S > O2S (first orientation); C5S′ > O2S′ (second orientation); C5SC > O2SC (third orientation). The sum of the occupancy factor of the three different orientations was constrained to be 0.5 using the SUMP instruction.

### Single-crystal X-ray crystallography of 1 → 2D MOF variant

Individual single-crystal samples were isolated from the substrate on which they were grown/formed via an oil-lubricated Kapton loop. All reflection intensities were measured at 110(2) K using a SuperNova diffractometer (equipped with Atlas detector) with Cu Kα radiation (*λ* = 1.54178 Å) under the program CrysAlisPro (Version CrysAlisPro 1.171.39.29c, Rigaku OD, 2017). The same program was used to refine the cell dimensions and for data reduction. The structure was solved with the program SHELXS-2014/7^45^ and was refined on *F*^2^with SHELXL-2014/7^45^. Analytical numeric absorption correction based on a multifaceted crystal model was applied using CrysAlisPro. The temperature of the data collection was controlled using the system Cryojet (manufactured by Oxford Instruments). The H atoms were placed at calculated positions using the instructions AFIX 43 with isotropic displacement parameters having values 1.2 Ueq of the attached C atoms. The structure is partly disordered. The crystallographic structure of the 2D MOF variant is provided in Supplementary Fig. 15.

The Mo–Mo complex is found at a site of twofold axial symmetry in the asymmetric unit, and only one half of the molecule is crystallographically independent. The crystal lattice includes some fair amount of lattice DMA solvent molecules that are found to be very disordered. In the final refinement, their contributions have been removed using the SQUEEZE procedure in Platon^46^.

### SEM

High resolution scanning electron micrographs were obtained on a Tescan Mira3 GMU SEM equipped with a field emission gun. Images of devices of **1** and **2** were collected using the Mira3’s in-lens secondary electron detector for beam energies between 10 and 20 kV, a working distance of 10 mm, and accumulation times of 10 min.

### Raman spectroscopy

Raman spectroscopy was performed on a Horiba-Jobin-Yvon T64000 Raman Spectrometer using a 514 nm laser operating at a power of 1 mW.

### Phase conversions

Conversion of **1** to **2** was initially observed by exposing CVD crystals to anhydrous DMA vapors. Crystals of **1** grown on glass substrates were placed within 20 mL glass scintillation vials, which were inserted, uncapped, within 100 mL VWR glass media bottles filled with 2 mL anhydrous DMA. The media bottles were capped and left undisturbed in RT conditions. Conversion to **2** could be observed in 48 h as an orange-red color change.

Cyclic conversions between **1** and **2** were more easily completed using bulk polycrystalline samples of each form. Solution grown crystals of **2** were obtained from recrystallization of 10 mg Mo_2_(INA)_4_ in 5 mL anhydrous DMA. Crystals grown after 48 h were collected and dried under vacuum. Five milligrams of crystals were placed in 20 mL glass scintillation vials, which were placed, uncapped, within 100 mL VWR glass media bottles filled with 2 mL anhydrous methanol for 48 h. Upon complete conversion to **1**, the remaining crystalline sample was placed in a second media bottle with 2 mL anhydrous DMA for 48 h. pXRD was used to monitor conversion between **1** and **2**.

### Heat capacity measurement

Heat capacity data were collected on a Quantum Design Physical Property Measurement System from 2 to 300 K, with a total of 80 temperature set points. Sample pellets were prepared by mechanically pressing powders of **1** converted from **2** then dried.

### Device fabrication

A single crystal of **1** was transferred from a growth substrate to a Si_3_N_4_/SiO_2_/Si substrate by mechanical exfoliation using Kapton tape. Electrical contacts (Ti (adhesion layer): 7 nm; Au (contact layer): 300 nm) were patterned over the MOF crystals by electron-beam lithography and then deposited through thermal evaporation. First, PMMA/MMA (C2/EL 11) layers were deposited over the device substrates containing the 1D and 2D crystals by spin-coating at 4000 rpm for 40 s. The resists were subjected to baking at 180 °C for 2 min after each coating step. The contact patterns were defined by electron-beam lithography (JEOL JSF-7001F) followed by resist development and rinsing in MIBK and IPA for 90 and 30 s, respectively. A 7-nm-thick Ti adhesion layer followed by a 300-nm-thick Au layer was deposited by thermal evaporation. Residual metal lift-off was performed in acetone over 3 h.

### Charge transport measurements

The fabricated devices were mounted to a *xyz*-translation stage, which is part of our homebuilt device characterization workstation. The devices were connected via Au-plated W probes (74JA-BT-PS108, American Probe & Technologies Inc.) and triax cables to an ultra-low noise (<100 fA) semiconductor parameter analyzer (Keithley 4200-SCS). The device current was recorded while the applied voltage was swept from –20 to 20 V. For the photocurrent measurement, the output of a continuous wave laser diode was focused on the device during the *I*–*V* measurement. The wavelength and spot size of the pump beam were 520 nm and ~1 µm, respectively. For characterization of the temporal photoresponse of the devices, periodic laser illumination was generated by using a homebuilt optical chopper with an interval and duration of 5 s.

### STM imaging

All STM experiments were performed in a homebuilt ultra-high-vacuum (UHV) STM system with Pt/Ir tips at *T* ~ 7 K. Standard Ar^+^ sputtering/annealing cycles were applied in order to prepare atomically-clean Au(111) growth substrates. Mo_2_(INA)_4_ precursors were deposited onto the Au(111) surface, which was held at room temperature under UHV conditions (base pressure ≈ 2 × 10^–10^ Torr), using a homebuilt Knudsen-type evaporator. The coverage was controlled by separately adjusting the evaporator temperature (typically 280 °C) and deposition time (typically 15 min). After deposition the sample was annealed up to 250 °C to form the single-layer 2D MOF. After growth the samples were directly transferred into the STM chamber and were cooled to ~7 K for STM imaging. All STM images were processed using WSxM freeware^47^.

## Supplementary information

Supplementary Information

## Data Availability

The data that support the findings of this study are available from the corresponding author (T.J.K.) upon reasonable request. The X-ray crystallographic coordinates for structures reported in this study have been deposited in the Cambridge Crystallographic Data Centre (CCDC), under deposition numbers CCDC-1870245 and CCDC-2031673.

## References

[1] Hendon CH, Rieth AJ, Korzyński MD, Dincă M (2017). Grand challenges and future opportunities for metal-organic frameworks. ACS Cent. Sci..

[2] Knebel A (2017). Defibrillation of soft porous metal-organic frameworks with electric fields. Science.

[3] Ko M, Mendecki L, Mirica KA (2018). Conductive two-dimensional metal-organic frameworks as multifunctional materials. Chem. Commun..

[4] Sun L, Campbell MG, Dincă M (2016). Electrically conductive porous metal-organic frameworks. Angew. Chem. Int. Ed..

[5] Medina DD, Mähringer A, Bein T (2018). Electroactive metalorganic frameworks. Isr. J. Chem..

[6] Solomos MA, Claire FJ, Kempa TJ (2019). 2D Molecular crystal lattices: advances in their synthesis, characterization, and application. J. Mater. Chem. A.

[7] Allendorf MD (2020). Electronic devices using open framework materials. Chem. Rev..

[8] Stassen I (2017). An updated roadmap for the integration of metal-organic frameworks with electronic devices and chemical sensors. Chem. Soc. Rev..

[9] Sato O (2016). Dynamic molecular crystals with switchable physical properties. Nat. Chem..

[10] Widmer RN (2019). Pressure promoted low-temperature melting of metal–organic frameworks. Nat. Mater..

[11] Sun L, Park SS, Sheberla D, Dincǎ M (2016). Measuring and reporting electrical conductivity in metal-organic frameworks: Cd2(TTFTB) as a case study. J. Am. Chem. Soc..

[12] Evans AM (2018). Seeded growth of single-crystal two-dimensional covalent organic frameworks. Science.

[13] Colson JW (2011). Oriented 2D covalent organic framework thin films on single-layer graphene. Science.

[14] Kumar, M., Choudhary, M. K. & Rimer, J. D. Transient modes of zeolite surface growth from 3D gel-like islands to 2D single layers. *Nat. Commun*. **9**, 1–9 (2018). 10.1038/s41467-018-04296-4 PMC5974312 29844357

[15] Xie LS (2018). Tunable mixed-valence doping toward record electrical conductivity in a three-dimensional metal-organic framework. J. Am. Chem. Soc..

[16] Talin AA (2014). Tunable electrical conductivity in metal-organic framework thin-film devices. Science.

[17] Bhardwaj SK (2018). An overview of different strategies to introduce conductivity in metal-organic frameworks and miscellaneous applications thereof. J. Mater. Chem. A.

[18] Calvo JJ, Angel SM, So MC (2020). Charge transport in metal-organic frameworks for electronics applications. APL Mater..

[19] Lee JM, Otake K, Kitagawa S (2020). Transport properties in porous coordination polymers. Coord. Chem. Rev..

[20] Xie LS, Skorupskii G, Dincă M (2020). Electrically conductive metal-organic frameworks. Chem. Rev..

[21] Huang X (2015). 2-dimensional π-d conjugated coordination polymer with extremely high electrical conductivity and ambipolar transport behaviour. Nat. Commun..

[22] Jin Z (2017). Solution-processed transparent coordination polymer electrode for photovoltaic solar cells. Nano Energy.

[23] Stassen I, De Vos D, Ameloot R (2016). Vapor-phase deposition and modification of metal–organic frameworks: State-of-the-art and future Directions. Chem. - A Eur. J..

[24] Stassin, T. et al. Vapour-phase deposition of oriented copper dicarboxylate metal–organic framework thin films. *Chem. Commun*. **5**, 5 (2019). 10.1039/c9cc05161a 31369024

[25] Krishtab M (2019). Vapor-deposited zeolitic imidazolate frameworks as gap-filling ultra-low-k dielectrics. Nat. Commun..

[26] Salmi LD (2013). Studies on atomic layer deposition of MOF-5 thin films. Microporous Mesoporous Mater..

[27] Lausund KB, Nilsen O (2016). All-gas-phase synthesis of UiO-66 through modulated atomic layer deposition. Nat. Commun..

[28] Ahvenniemi E, Karppinen M (2016). Atomic/molecular layer deposition: a direct gas-phase route to crystalline metal-organic framework thin films. Chem. Commun..

[29] Stassen I (2016). Chemical vapour deposition of zeolitic imidazolate framework thin films. Nat. Mater..

[30] Li MM (2019). Molecular chains of coordinated dimolybdenum isonicotinate paddlewheel clusters. RSC Adv..

[31] Claire FJ (2018). Hierarchically ordered two-dimensional coordination polymers assembled from redox-active dimolybdenum clusters. J. Am. Chem. Soc..

[32] Chisholm MH, Macintosh AM (2005). Linking multiple bonds between metal atoms: clusters, dimers of ‘dimers’, and higher ordered assemblies. Chem. Rev..

[33] Chisholm MH (2007). Metal to metal multiple bonds in ordered assemblies. Proc. Natl Acad. Sci. USA.

[34] Cotton FA (2003). Fully localized mixed-valence oxidation products of molecules containing two linked dimolybdenum units: An effective structural criterion. J. Am. Chem. Soc..

[35] Chisholm MH, Lear BJ (2011). M2δ to ligand π-conjugation: testbeds for current theories of mixed valence in ground and photoexcited states of molecular systems. Chem. Soc. Rev..

[36] Kubiak CP (2013). Inorganic electron transfer: sharpening a fuzzy border in mixed valency and extending mixed valency across supramolecular systems. Inorg. Chem..

[37] Cotton FA, Donahue JP, Murillo CA (2002). The first supramolecular assemblies comprised of dimetal units and chiral dicarboxylates. Inorg. Chem. Commun..

[38] Cotton FA, Lin C, Murillo CA (2001). Supramolecular arrays based on dimetal building units. Acc. Chem. Res..

[39] Cotton FA, Mester ZC, Webb TR (1974). Dimolybdenum tetraacetate. Acta Crystallogr. Sect. B.

[40] Hmadeh M (2012). New porous crystals of extended metal-catecholates. Chem. Mater..

[41] Park SS (2015). Cation-dependent intrinsic electrical conductivity in isostructural tetrathiafulvalene-based microporous metal-organic frameworks. J. Am. Chem. Soc..

[42] Wang Q-X (2010). Rigid pillars and double walls in a porous metal-organic framework: single-crystal to single-crystal, controlled uptake and release of iodine and electrical conductivity. J. Am. Chem. Soc..

[43] Day (2019). Single crystals of electrically conductive two-dimensional metal–organic frameworks: structural and electrical transport properties. ACS Cent. Sci..

[44] Nečas D, Klapetek P (2012). Gwyddion: an open-source software for SPM data analysis. Cent. Eur. J. Phys..

[45] Sheldrick GM (2015). Crystal structure refinement with SHELXL. Acta Crystallogr. Sect. C. Struct. Chem..

[46] Spek AL (2009). Structure validation in chemical crystallography. Acta Crystallogr. Sect. D. Biol. Crystallogr..

[47] Horcas I (2007). WSXM: a software for scanning probe microscopy and a tool for nanotechnology. Rev. Sci. Instrum..

