# Supplementary material for: Structural and electronic switching of a single crystal 2D metal-organic framework prepared by chemical vapor deposition
Source: Nat Commun. 2020 Nov 2;11:5524. doi: 10.1038/s41467-020-19220-y (PMC7608636; doi:10.1038/s41467-020-19220-y)
Supplement: Supplementary file 1 — Supplementary Information [file 41467_2020_19220_MOESM1_ESM.pdf]

Supplementary Information for

Structural and electronic switching of a single crystal 2D metal-organic framework prepared by chemical vapor deposition

F. James Claire[1†], Marina A. Solomos[1†], Jungkil Kim[1], Gaoqiang Wang[2,3], Maxime A. Siegler[1], Michael F. Crommie[2,3,4], and Thomas J. Kempa[1,5]*

[1]Department of Chemistry, Johns Hopkins University, Baltimore, MD, USA.

[2]Department of Physics, University of California Berkeley, Berkeley, California, USA

[3]Materials Sciences Division, Lawrence Berkeley National Laboratory, Berkeley, California, USA

[4]Kavli Energy NanoSciences Institute at the University of California Berkeley and the Lawrence Berkeley National Laboratory, Berkeley, California, USA

[5]Department of Materials Science and Engineering, Johns Hopkins University, Baltimore, MD, USA.

† These authors contributed equally to this work.

* Correspondence to Thomas J. Kempa (tkempa@jhu.edu)

Supplementary Information Content	*Page(s)*
**Supplementary Fig. 1:** CVD growth of **1** at various conditions	S3
**Supplementary Fig. 2:** Atomic force microscopy of **1** (single crystal)	S4
**Supplementary Fig. 3:** Atomic force microscopy of **1** (layers)	S5
**Supplementary Fig. 4:** Crystal structure 1D zig-zag chain	S6
**Supplementary Table 1:** Crystallographic information for **1** and **2**	S7–8
**Supplementary Fig. 5:** pXRD and BFDH morphology of **1**	S9
**Supplementary Fig. 6:** Bond distances and body diagonals of **1**	S10
**Supplementary Fig. 7:** Single crystal structure of **2**	S11
**Supplementary Fig. 8:** Bond distances and body diagonals of **2**	S12
**Supplementary Table 2:** Crystallographic data of 2D MOF variant	S13
**Supplementary Fig. 9:** Heat capacity data for **1**	S14
**Supplementary Fig. 10:** Single crystal structure of 2D MOF variant	S15
**Supplementary Fig. 11:** Dimensions of devices of **1** and **2**	S16
**Supplementary Fig. 12:** STM maps of single layer $Mo_2(INA)_4$ on Au(111)	S17
**Supplementary Fig. 13:** Structural figure of **1**	S18
**Supplementary Fig. 14:** Structural figure of **2**	S19
**Supplementary Fig. 15:** Structural figure of 2D MOF variant	S20

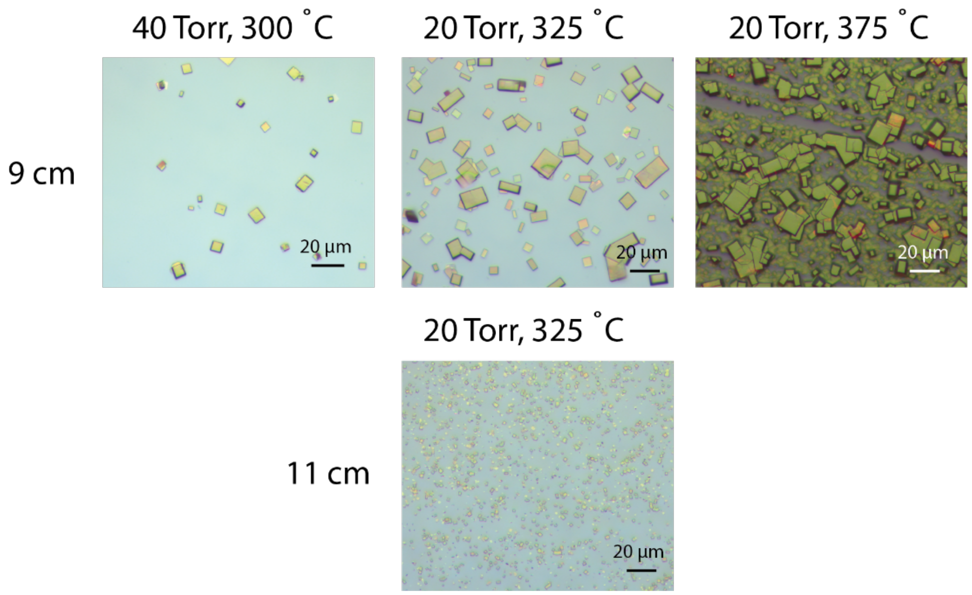

Supplementary Fig. 1 | CVD growth of 1 at various conditions. Optical images of representative crystal growth under various growth conditions. Crystals grown at 20 Torr and 325 °C have edge lengths up to ~50 µm and have a mean area of 96 ± 55 μm^2 (center). Crystals grown at lower temperatures are typically smaller in size and exhibit lower nucleation density (left) while crystals grown at higher temperatures exhibit greater surface coverage of the deposition substrate and begin to form films (right). Crystals grown with the substrate 11 cm downstream of the precursor boat are smaller and thinner (bottom).

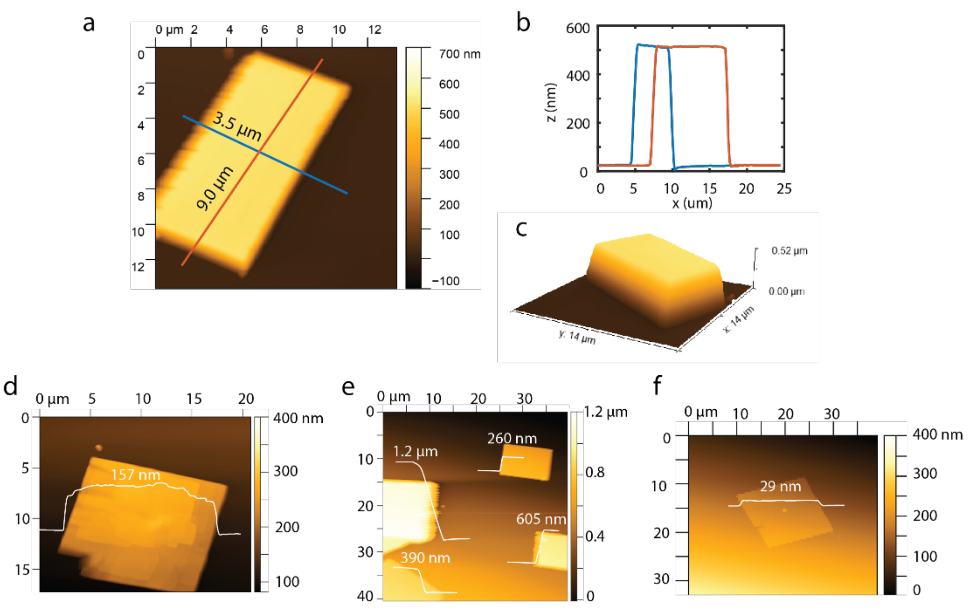

Supplementary Fig. 2 | Atomic force microscopy of 1 (single crystal). a, Atomic force micrograph of a 9.0 µm × 3.5 µm rectangular crystal of **1**. **b**, Line profiles taken along the annotated portions of the AFM in **a**. The crystal is uniform and 500 nm thick. **c**, 3D AFM map of the crystal displayed in **a**. **d**,**e**, Atomic force micrographs of crystals synthesized under standard reaction conditions with the substrate 9 cm downstream of the precursor. These crystals have thicknesses in the range of 100 – 1200 µm. **f,** Atomic force micrograph of a single crystalline deposit synthesized under standard reaction conditions with the substrate 11 cm downstream of the precursor. The crystal is 29 µm thick.

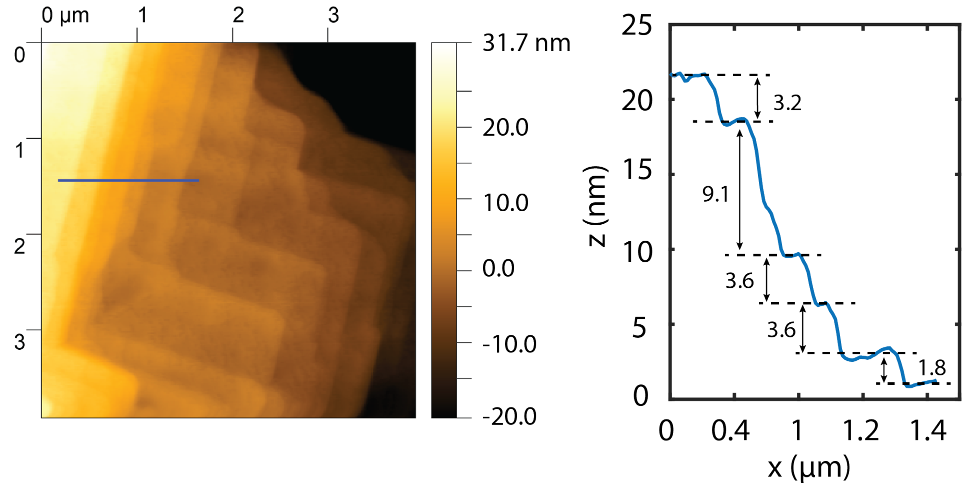

Supplementary Fig. 3 | Atomic force microscopy of 1 (layers). Atomic force micrograph of the edge of a crystal of **1**. Step edges are clearly visible and are associated with layers measuring between 1.8 nm and 9.1 nm. These layers are likely comprised of between 2 and 10 stacked sheets of horizontally assembled 1D zig-zag chains as shown in Fig. 2g.

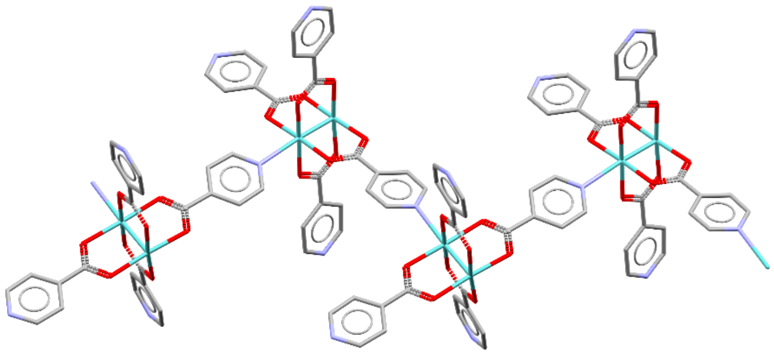

Supplementary Fig. 4 | Crystal structure 1D zig-zag chain. Crystal packing structure of an under-coordinated 1D chain of **1**. H atoms and disorder have been omitted for clarity.

Supplementary Table 1 | Crystallographic information of 1 and 2

	CVD MoINA (**1**) xs1592a	2D MoINA (**2**) xs1751a
Crystal data		
Chemical formula	$C_{24}H_{16}Mo_2N_4O_8$	$C_{24}H_{16}Mo_2N_4O_8 \cdot 2(C_4H_9NO)$
M_r	680.29	854.53
Crystal system, space group	Orthorhombic, *Pbca*	Monoclinic, *P*2/*c*
Temperature (K)	175	110
a, *b*, *c* (Å)	14.8962 (3), 14.8796 (3), 26.2130 (4)	16.3096 (7), 15.3613 (4), 14.4440 (4)
β (°)	90°	93.000 (3)
V (Å^3)	5810.10 (19)	3613.8 (2)
Z	8	4
Radiation type	Cu *K*α	Cu *K*α
μ (mm^{-1})	7.50	6.22
Crystal size (mm)	0.07 × 0.07 × 0.02	0.05 × 0.04 × 0.01
Data collection		
Diffractometer	SuperNova, Dual, Cu at zero, Atlas	SuperNova, Dual, Cu at zero, Atlas
Absorption correction	Analytical *CrysAlis PRO* 1.171.39.29c (Rigaku Oxford Diffraction, 2017) Analytical numeric absorption correction using a multifaceted crystal model based on expressions derived by R.C. Clark & J.S. Reid. (Clark, R. C. & Reid, J. S. (1995). Acta Cryst. A51, 887-897) Empirical absorption correction using spherical harmonics, implemented in SCALE3 ABSPACK scaling algorithm.	Gaussian *CrysAlis PRO* 1.171.39.29c (Rigaku Oxford Diffraction, 2017) Numerical absorption correction based on gaussian integration over a multifaceted crystal model Empirical absorption correction using spherical harmonics, implemented in SCALE3 ABSPACK scaling algorithm.
T_{min}, T_{max}	0.648, 0.876	0.795, 0.941
No. of measured, independent and observed [$I > 2\sigma(I)$] reflections	19400, 5697, 4758	21755, 7093, 4009
R_{int}	0.034	0.087
$(\sin \theta/\lambda)_{max}$ (Å^{-1})	0.617	0.617

Refinement		
$R[F^2 > 2\sigma(F^2)]$, $wR(F^2)$, S	0.044, 0.114, 1.05	0.057, 0.151, 1.01
No. of reflections	5697	7093
No. of parameters	407	623
No. of restraints	304	596
H-atom treatment	H-atom parameters constrained	H-atom parameters constrained
$\Delta\rho_{max}$, $\Delta\rho_{min}$ (e Å^{-3})	1.23, -1.05	1.50, -0.87

Computer programs: *CrysAlis PRO* 1.171.39.29c (Rigaku OD, 2017), *SHELXS2014*/7 (Sheldrick, 2015), *SHELXL2014*/7 (Sheldrick, 2015), *SHELXTL* v6.10 (Sheldrick, 2008).

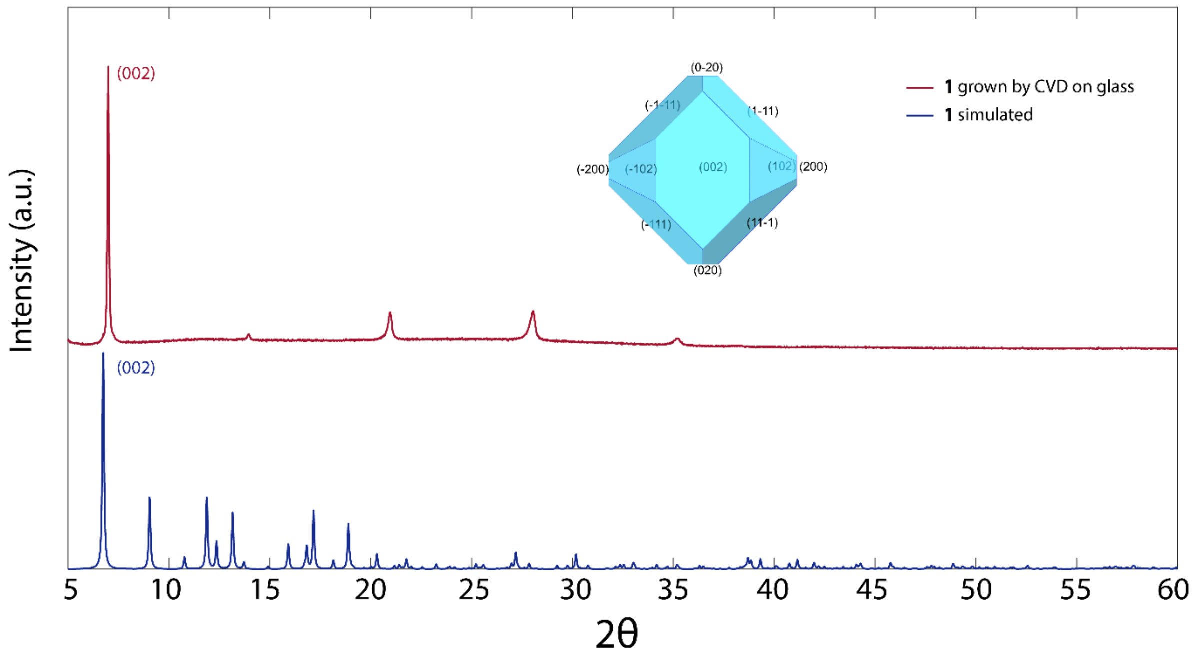

Supplementary Fig. 5 | pXRD and BFDH morphology of 1. pXRD of **1** grown on glass substrate (red) compared to a simulated pXRD pattern of **1** from its single crystal structure (blue). The intense peak at ~6.7° agrees well with the (002) reflection of the simulated diffraction pattern. The BFDH morphology calculated from Mercury also suggests that the crystals grow with the large (002) crystal face parallel to the glass substrate surface.

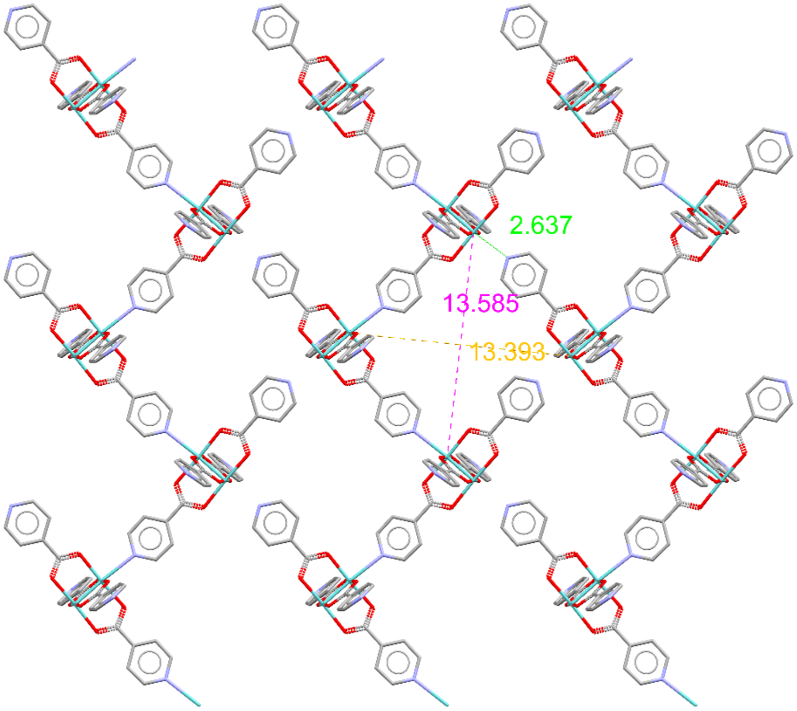

Supplementary Fig. 6 | Bond distances and body diagonals of 1. Body diagonals and Mo–N distances of **1**. The Mo–N distance of 2.637 Å is longer than the 2.546(3) Å and 2.586(3) Å Mo–N bond lengths previously reported in the 2D phase. The measured body diagonals are in agreement with those reported for the square sub-cells of the 2D phase.

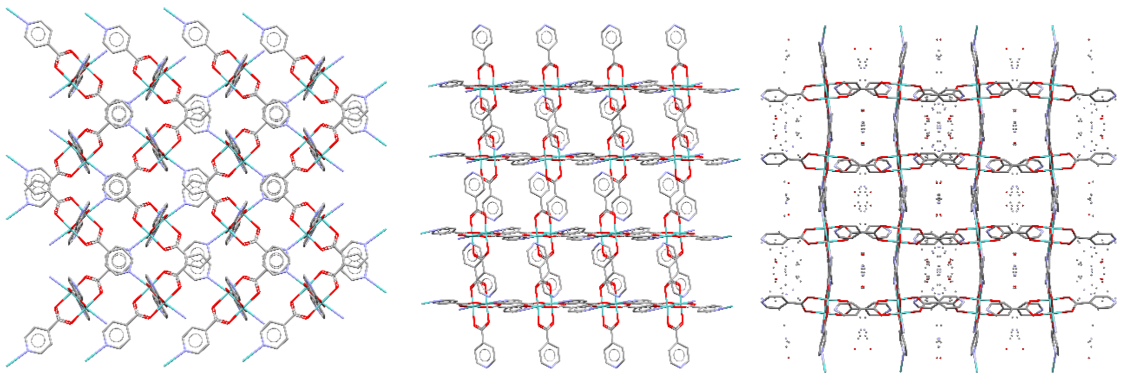

Supplementary Fig. 7 | Single crystal structure of 2. Crystal packing structure of **2** viewed down the *a* (left), *b* (center), and *c* (right) axes. Disordered DMA molecules are included in the view down the *c* axis to show solvent arrangement within the framework. We note that **1** has a void space of 350 Å^3 which corresponds to 6% of its unit cell (measured using a 2.0 Å probe radius), and that this space is consistent with uptake of DMA molecules required for conversion of **1** to **2**. Though bulk and single crystal analyses repeatedly yielded the structure reported in this figure, we did observe on one occasion a closely-related variant of this 2D MOF. Details of this 2D MOF variant are provided in Supplementary Fig. 10.

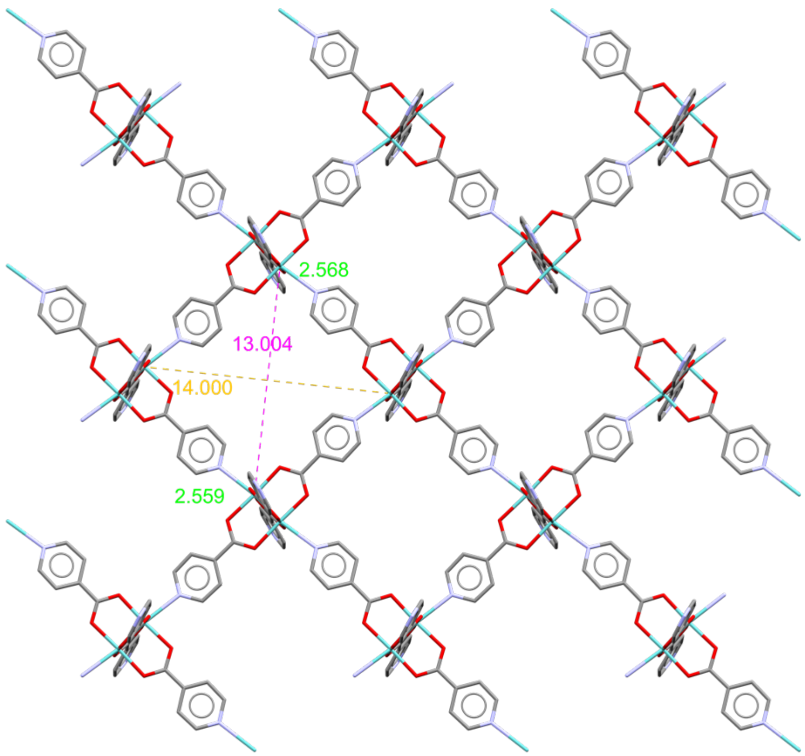

Supplementary Fig. 8 | Bond distances and body diagonals of 2. Body diagonals and Mo–N distances of **2**. The Mo–N bond lengths of 2.568 Å and 2.559 Å agree well with the 2.546(3) Å and 2.586(3) Å Mo–N bond lengths previously reported for the 2D phase and are also shorter than the 2.637 Å Mo–N distance reported for **1**. The measured body diagonals are also in agreement with those reported for the square sub-cells of the 2D phase.

Supplementary Table 2 | Crystallographic data of 2D MOF variant

	xs1767a
Crystal data	
Chemical formula	$C_{24}H_{16}Mo_2N_4O_8$
M_r	680.29
Crystal system, space group	Monoclinic, $C2/c$
Temperature (K)	110
a, b, c (Å)	16.4587 (2), 15.3102 (2), 14.3911 (2)
β (°)	95.7337 (15)
V (Å^3)	3608.21 (8)
Z	4
Radiation type	Cu $K\alpha$
μ (mm^{-1})	6.04
Crystal size (mm)	0.07 × 0.06 × 0.02
Data collection	
Diffractometer	SuperNova, Dual, Cu at zero, Atlas
Absorption correction	Analytical<br>*CrysAlis PRO* 1.171.39.29c (Rigaku Oxford Diffraction, 2017) Analytical numeric absorption correction using a multifaceted crystal model based on expressions derived by R.C. Clark & J.S. Reid. (Clark, R. C. & Reid, J. S. (1995). Acta Cryst. A51, 887-897) Empirical absorption correction using spherical harmonics, implemented in SCALE3 ABSPACK scaling algorithm.
T_{min}, T_{max}	0.708, 0.910
No. of measured, independent and observed [I > 2[illegible](I)] reflections	10962, 3532, 3087
R_{int}	0.030
(sin θ/λ)$_{max}$ (Å^{-1})	0.616
Refinement	
$R[F^2 > 2\sigma(F^2)]$, $wR(F^2)$, S	0.031, 0.086, 1.06
No. of reflections	3532
No. of parameters	172
H-atom treatment	H-atom parameters constrained
$\Delta\rho_{max}$, $\Delta\rho_{min}$ (e Å^{-3})	1.39, -0.51

Computer programs: *CrysAlis PRO* 1.171.39.29c (Rigaku OD, 2017), *SHELXS2014*/7 (Sheldrick, 2015), *SHELXL2014*/7 (Sheldrick, 2015), *SHELXTL* v6.10 (Sheldrick, 2008).

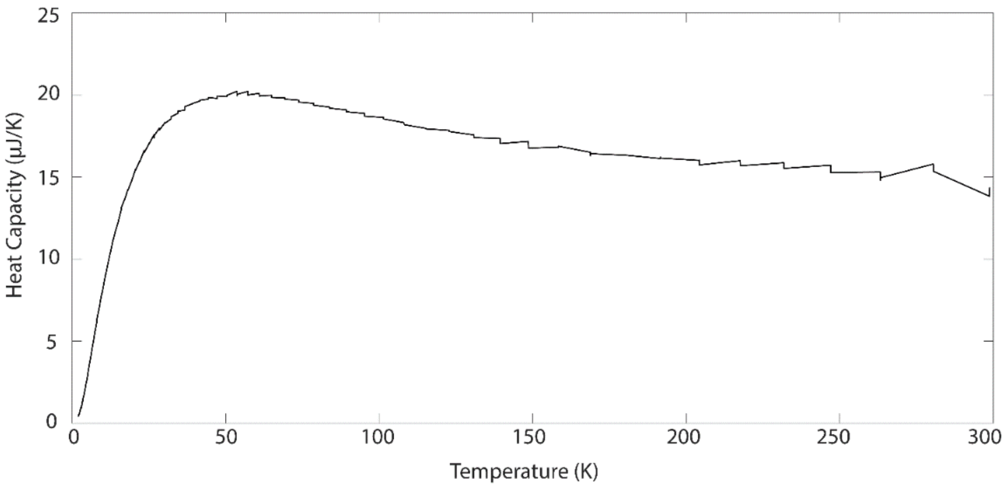

Supplementary Fig. 9 | Heat capacity data for 1. Heat capacity data for **1** after its conversion from **2** in the presence of methanol vapor. A 5.2 mg pellet was prepared from a dried sample of **1** and then analyzed from 2 K to 300 K. These data suggest this converted phase is stable at room temperature, an observation corroborated by our pXRD results.

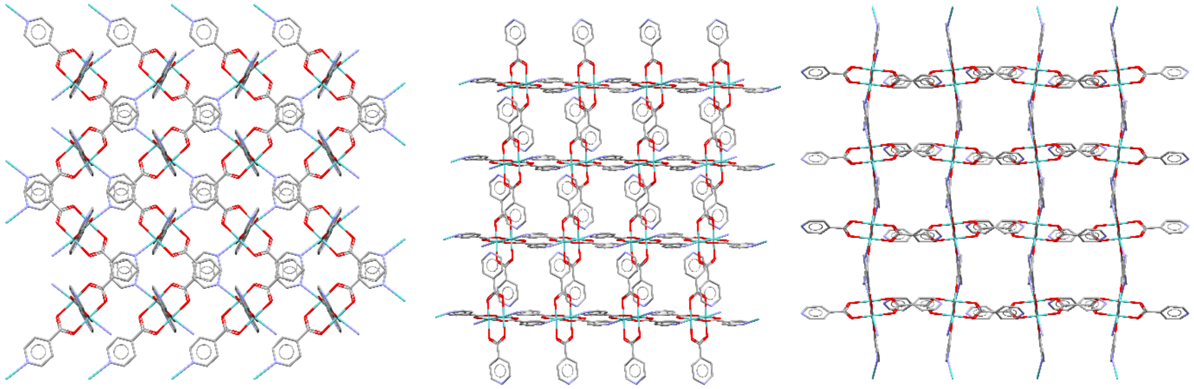

Supplementary Fig. 10 | Single crystal structure of 2D MOF variant. Crystal packing of an additional 2D phase viewed down the *a* (left), *b* (center), and *c* (right) axes. These structure data, though significantly similar to **2**, suggest that solvent inclusion and intrinsic framework flexibility may facilitate the formation of assorted 2D phases.

1

2

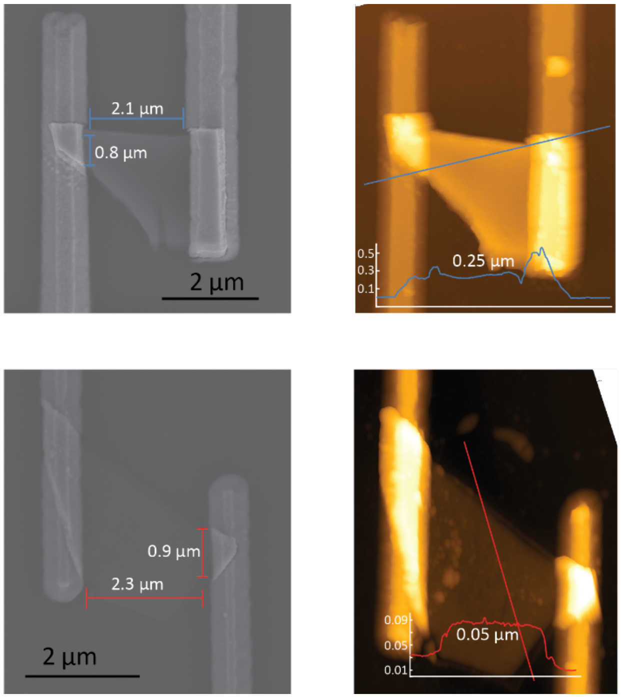

Supplementary Fig. 11 | Dimensions of devices of 1 and 2. SEM and AFM images of a device fabricated from exfoliated **1** (top) and **2** (bottom) crystals. The dimensions indicated here were used, in conjunction with measured current values (Fig. 4b,c) to calculate the conductivity of each device.

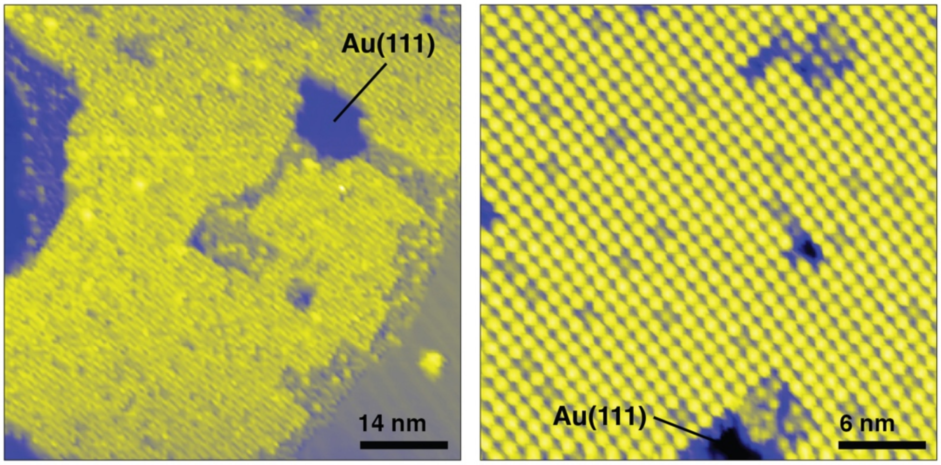

Supplementary Fig. 12 | STM maps of single layer $Mo_2(INA)_4$ on Au(111). STM maps collected after sample annealing. Cluster dose was adjusted to provide a 0.8 ML coverage. Under these conditions, bare Au(111) regions are visible and serve as a reference against which we verify that our 2D MOF is a single layer. The 2D MOF clearly persists over the Au(111) surface. *Imaging parameters*: V_{bias} = 0.05 V, I_{tunnel} = 5 pA.

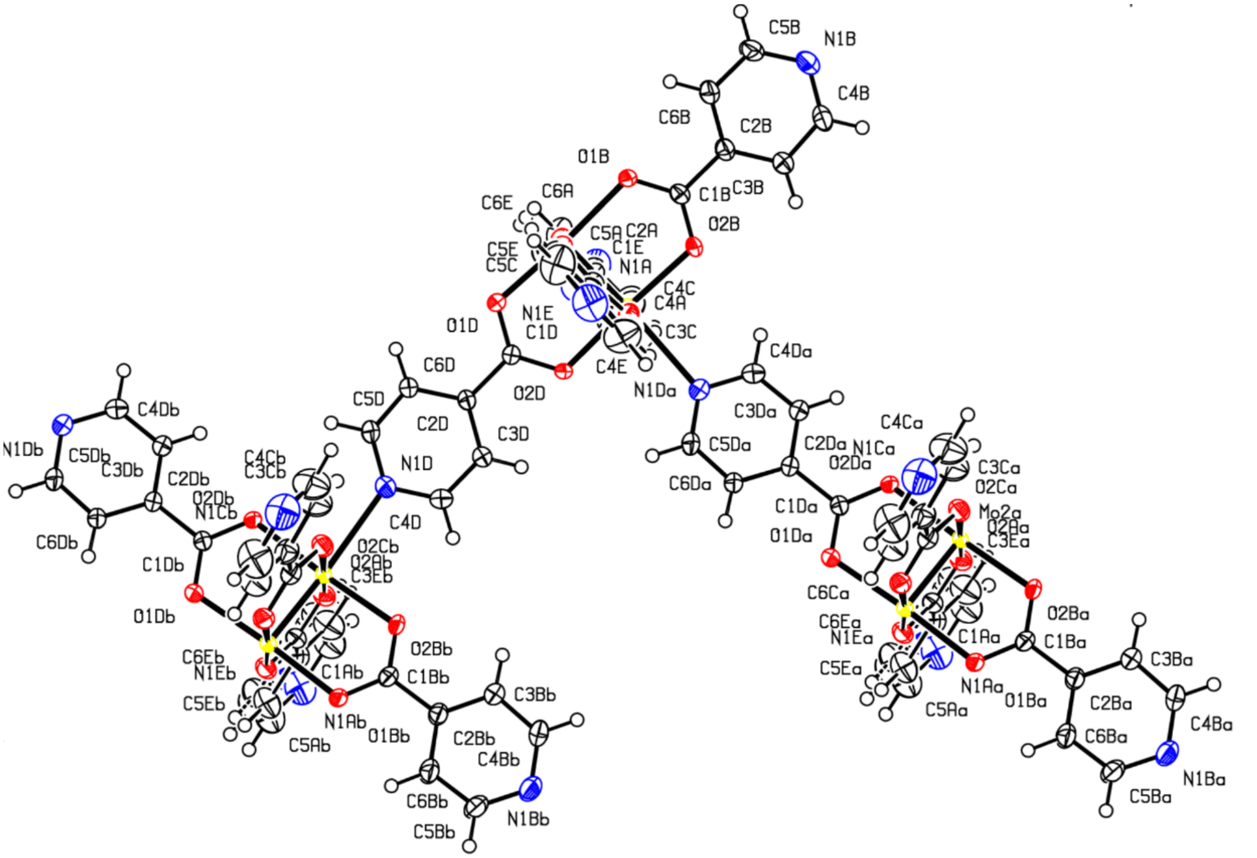

Supplementary Fig. 13 | Structural figure of 1. Crystallographic structure of **1** with ellipsoids set at 50% probability. See Supplementary Table 1 (xs1592a) and associated CIF file for more information.

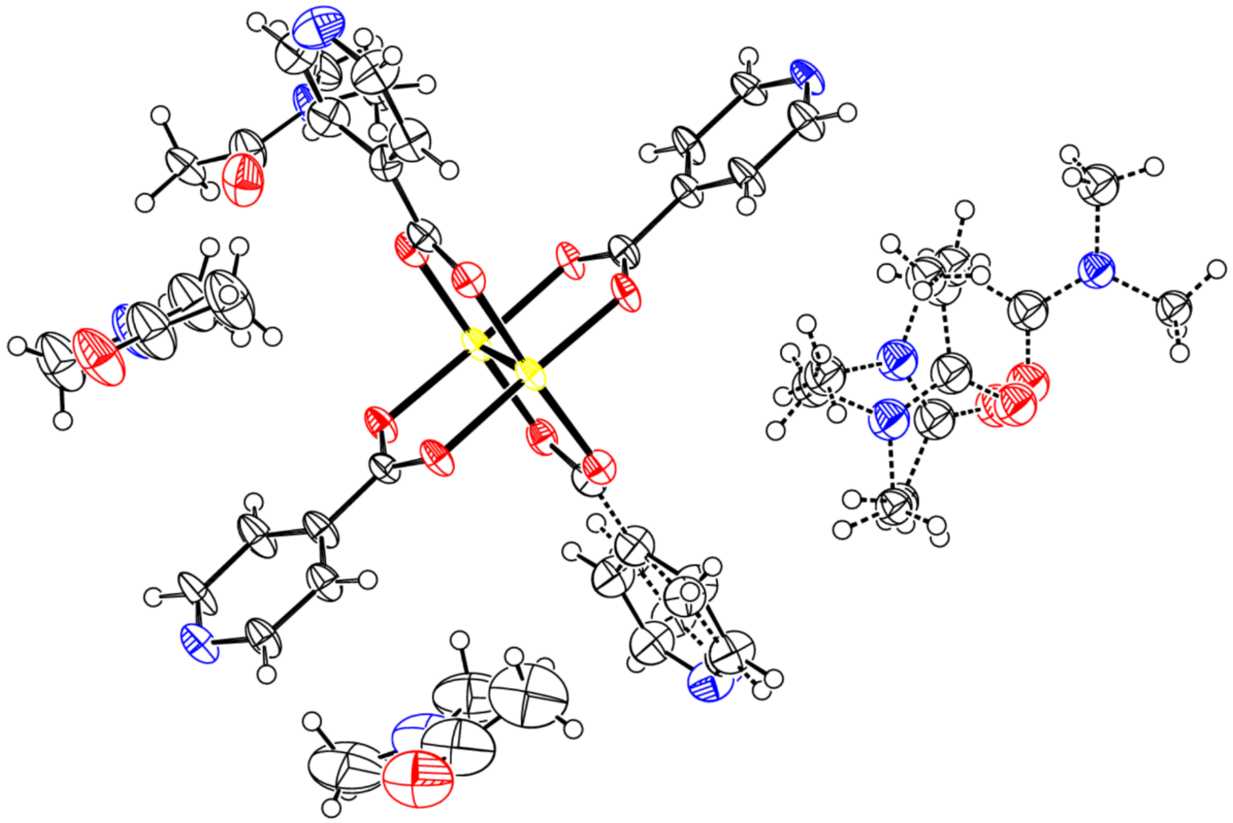

Supplementary Fig. 14 | Structural figure of 2. Crystallographic structure of **2** with ellipsoids set at 50% probability. See Supplementary Table 1 (xs1751a) and associated CIF file for more information.

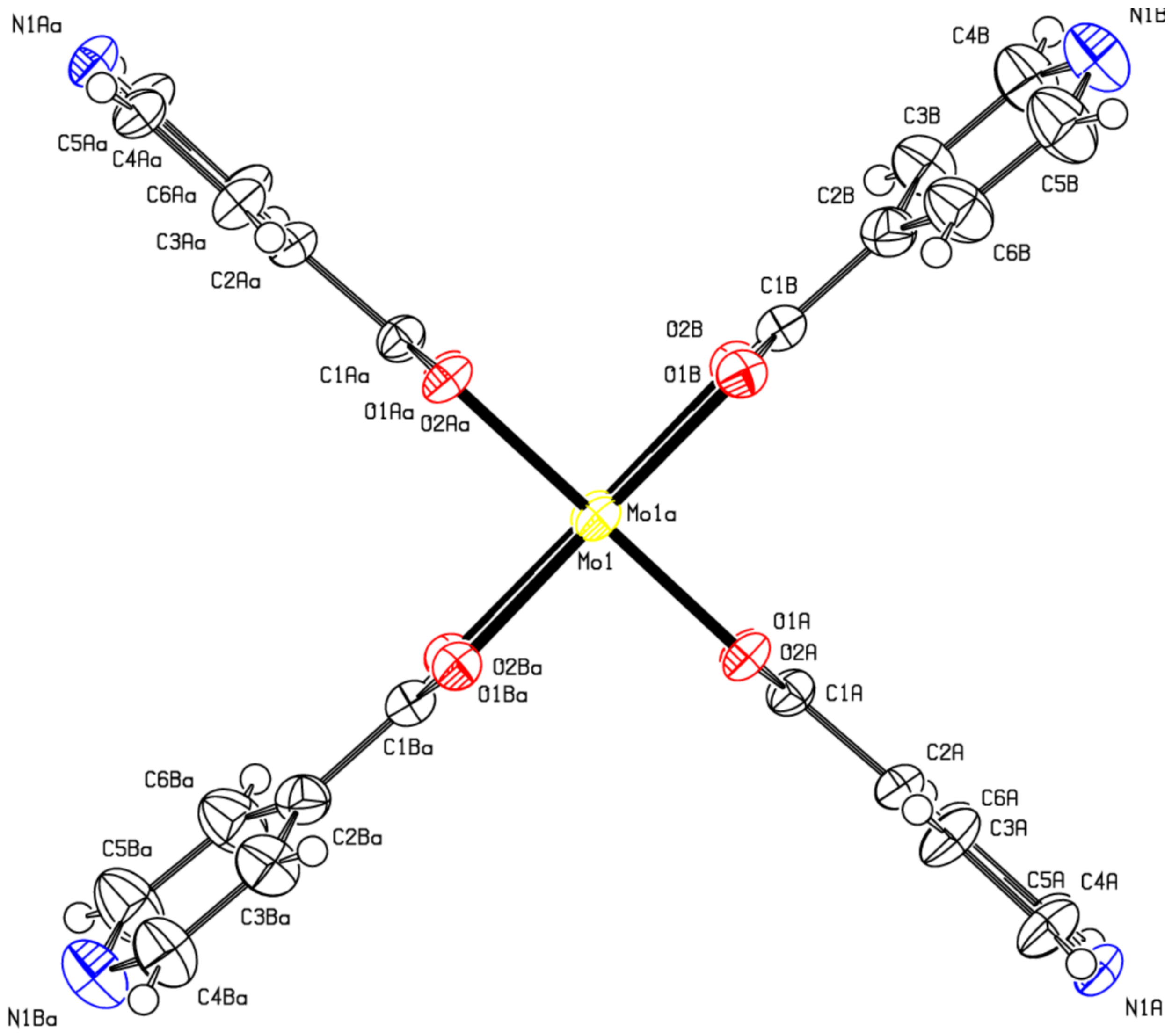

Supplementary Fig. 15 | Structural figure of 2D MOF variant. Crystallographic structure of 2D MOF variant with ellipsoids set at 50% probability. See Supplementary Table 2 (xs1767a) and associated CIF file for more information.
